# A Cross-Attention-Based Class Alignment Network for Cross-Subject EEG Classification in a Heterogeneous Space

**DOI:** 10.3390/s24217080

**Published:** 2024-11-03

**Authors:** Sufan Ma, Dongxiao Zhang

**Affiliations:** School of Science, Jimei University, Xiamen 361000, China; m-sf@jmu.edu.cn

**Keywords:** brain–computer interface, cross-subject EEG classification, domain adaptation, heterogeneous spaces, deep learning

## Abstract

Background: Domain adaptation (DA) techniques have emerged as a pivotal strategy in addressing the challenges of cross-subject classification. However, traditional DA methods are inherently limited by the assumption of a homogeneous space, requiring that the source and target domains share identical feature dimensions and label sets, which is often impractical in real-world applications. Therefore, effectively addressing the challenge of EEG classification under heterogeneous spaces has emerged as a crucial research topic. Methods: We present a comprehensive framework that addresses the challenges of heterogeneous spaces by implementing a cross-domain class alignment strategy. We innovatively construct a cross-encoder to effectively capture the intricate dependencies between data across domains. We also introduce a tailored class discriminator accompanied by a corresponding loss function. By optimizing the loss function, we facilitate the aggregation of features with corresponding classes between the source and target domains, while ensuring that features from non-corresponding classes are dispersed. Results: Extensive experiments were conducted on two publicly available EEG datasets. Compared to advanced methods that combine label alignment with transfer learning, our method demonstrated superior performance across five heterogeneous space scenarios. Notably, in four heterogeneous label space scenarios, our method outperformed the advanced methods by an average of 7.8%. Moreover, in complex scenarios involving both heterogeneous label spaces and heterogeneous feature spaces, our method outperformed the state-of-the-art methods by an average of 4.1%. Conclusions: This paper presents an efficient model for cross-subject EEG classification under heterogeneous spaces, which significantly addresses the challenges of EEG classification within heterogeneous spaces, thereby opening up new perspectives and avenues for research in related fields.

## 1. Introduction

Brain–computer interfaces (BCIs), a novel communication tool based on the brain’s neural activity, decode neuronal activity in the human brain into specific computer commands, thereby realizing direct communication between the human brain and electronic devices [1,2]. Electroencephalography (EEG) signals, which capture neuronal activity in the human brain by measuring voltage fluctuations on the scalp, have emerged as the most frequently utilized input signals for BCIs due to their convenience, safety, and cost-effectiveness. They have demonstrated promising performance in numerous BCI applications, including emotion recognition [3,4], epilepsy detection [5,6], and driver fatigue monitoring [7,8].

Motor imagery (MI), as one of the most popular BCI paradigms, has been widely applied in various fields, including robotic wheelchairs [9], spelling for the disabled [10,11,12], and neural rehabilitation [13,14]. A complete MI-based BCI system typically consists of five components: signal acquisition, signal processing, feature extraction and design, classification, and device control. To enhance the overall system efficiency, researchers have devoted considerable effort to designing distinguishable features and training efficient classification models. Consequently, this paper also emphasizes the critical aspects of signal processing, feature extraction, and design, as well as classification.

In the early stages of traditional methodologies, the extraction of EEG features was heavily reliant on human expertise and specific algorithms. Among these, Common Spatial Pattern (CSP) [15] emerged as the most frequently utilized spatial filtering approach, with Filter Bank Common Spatial Pattern (FBCSP) [16] being the most exemplary. Additionally, to gain deeper insights into the time–frequency characteristics of EEG signals, techniques such as the Continuous Wavelet Transform (CWT) [17] and Empirical Wavelet Transform (EWT) [18,19] were frequently adopted, providing powerful tools for EEG analysis. These meticulously extracted EEG features were then input into various traditional classifiers, including linear discriminant analysis (LDA) [20,21] and support vector machine (SVM) [22,23], for subsequent recognition and classification tasks.

In recent years, deep learning methods have gradually been incorporated into EEG decoding, achieving impressive results due to their powerful feature extraction capabilities [24,25]. Unlike traditional machine learning approaches, the training of deep neural networks necessitates a substantial amount of data. To fully leverage the known data from other subjects, researchers have proposed transfer learning (TL) [26] or domain adaptation (DA). Its core idea revolves around utilizing data from auxiliary subjects (i.e., source subjects or source domain) to enhance the learning performance of new subjects (i.e., target subjects or target domain). This is achieved through two primary strategies: data alignment, which aims to map data from both the source and target domains into a common space for seamless integration [27,28], and feature alignment, which involves constructing complex deep network architectures to precisely extract features and ensure, through the overall coordination of the network, a high degree of consistency between the features from both domains in the feature space.

**Figure 1:**
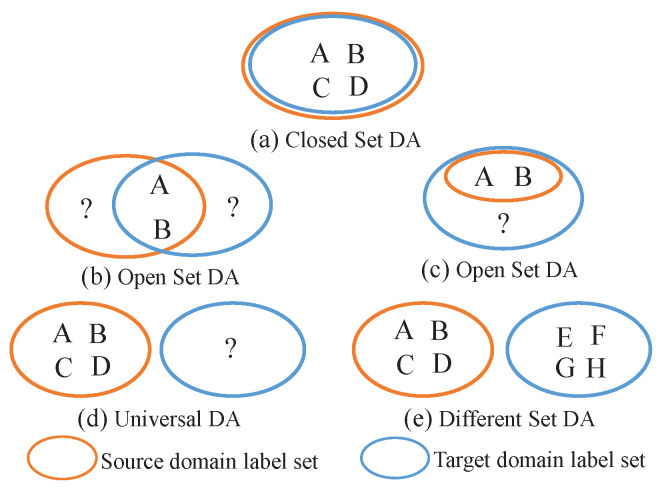
Different DA scenarios. A, B, …, H represent different classes. “?” represents unknown classes.

However, it is noteworthy that the majority of the current DA methods are centered on the scenario of homogeneous TL, which presupposes that the source and target domains share an identical feature space and label space, as illustrated in Figure 1a. This assumption often fails to hold in real-world applications, particularly in the field of BCIs, where the challenge of heterogeneous spaces is particularly pronounced.

On the one hand, heterogeneous feature spaces primarily refer to the situation in which the source and target subjects may utilize different electrode cap configurations, leading to significant variations in the number and positions of the channels in the collected EEG data. This poses a formidable challenge, and there are currently relatively few studies on heterogeneous TL. Wu et al. [29] attempted to address this issue by selecting source domain channel data that were closely aligned with the target domain channels. Wu and Xie et al. [30] proposed a new model named Informative Representation Fusion (IRF), which addresses the unsupervised heterogeneous DA problem by leveraging hypergraphs to handle the high-order correlations in non-independent identically distributed data, combining multi-layer perceptron networks to process independent identically distributed data, fusing these two representations through an attention mechanism, and aligning the distributions of the source and target domains based on the fused features using the maximum mean discrepancy.

On the other hand, heterogeneous label spaces typically arise when the source and target subjects perform different types of MI tasks, resulting in distinct labels for the EEG data collected from the two domains. This can manifest in various scenarios, as illustrated in Figure 1b–e. Busto and Gall [31] were the first to introduce the concept of open-set DA, as illustrated in Figure 1b, which postulates that the source and target domains share some known classes but also possess unique, unknown classes of their own. Teng et al. [32] proposed another scenario where the target domain encompasses all classes present in the source domain, in addition to some “unknown” classes, as illustrated in Figure 1c. Furthermore, the notion of universal DA has been suggested [33], as illustrated in Figure 1d, wherein, if a target domain sample can be categorized into any of the known classes in the source domain, it is classified accordingly; otherwise, it is labeled as “unknown”. Wu et al. [28] took into account the scenario of different types of DA, as illustrated in Figure 1e, where the target domain contains partially or completely different classes from the source domain. This paper is based on discussions of this particular scenario.

In addressing the challenges of DA in heterogeneous spaces, this paper presents an innovative end-to-end deep learning network architecture. Specifically, we construct a generator module capable of efficiently extracting high-dimensional features from EEG signals. To further exploit the intrinsic value of these features, we innovatively integrate self-attention and cross-attention mechanisms, pioneering the design of a cross-encoder. This encoder not only performs self-attention operations within individual domains to capture internal data dependencies but also enables cross-attention interactions between different domains, effectively facilitating the alignment and fusion of marginal probability distributions across different domains. Subsequently, we introduce a tailored class discriminator and corresponding loss function, which further enhance the model’s accuracy in cross-subject classification tasks by minimizing the differences between corresponding classes. Ultimately, we construct a set of parallel classifiers that independently perform classification tasks, collectively enhancing the precision and efficiency of the overall classification task.

The main contributions of this paper can be summarized as follows.

1. We propose a deep learning network framework tailored to DA classification problems in heterogeneous spaces, demonstrating notable advantages and potential in the field of EEG signal processing.

2. We innovatively design a cross-encoder based on the integration of self-attention and cross-attention mechanisms, achieving the combination of intra-domain self-attention computation and cross-attention computation between different domains.

## 2. Related Works

### 2.1. Transfer Learning

Recently, DA methods have demonstrated promising performance in addressing cross-subject issues [34,35,36,37,38], leveraging sufficient annotated EEG data from other subjects to model target subjects at both the data and feature levels, with the aim of minimizing the distribution discrepancy between the source and target domains.

In the realm of data alignment, He and Wu [27] innovatively proposed a low-cost computational approach capable of effectively aligning EEG data from diverse subjects within a Euclidean space, ultimately enhancing the classification performance for new subjects. Meanwhile, another study [28] adopted a supervised strategy, constructing a dedicated labeled sample set for each class of the target subject. The core objective of this method lies in minimizing the discrepancy between samples of a similar class across different domains, thereby optimizing the classification outcomes.

In the realm of feature alignment, Raza et al. [39] described a method for the detection of covariate shifts in extracted features, adapting the classifier accordingly. Jeon et al. [40] meticulously selected a source subject that demonstrated comparable characteristics to the target subject based on the power spectral density (PSD) features of their EEG signals during the resting state. Subsequently, the EEG signals from both the source and target subjects were integrated and jointly utilized in the training process of a deep neural network. Zhang et al. [41] introduced the Conditional Domain Adaptation Neural Network (CDAN) framework, aiming to decode MI-EEG signals and learn cross-subject common EEG feature representations. In this way, models trained on source subjects can effectively assist in the classification tasks of target subject signals. Zhao et al. [38] proposed an innovative Deep Representation Domain Adaptation (DRDA) network model, which employs a domain discriminator mechanism to achieve the precise alignment of deep features between two different domains.

It is noteworthy that, with the exception of the method in [28], the aforementioned approaches all belong to the category of homogeneous TL methods. The issue of heterogeneous TL in the field of EEG remains an open challenge that awaits further exploration.

### 2.2. Self-Attention Mechanism

The self-attention mechanism enables models to capture long-range dependencies [42]. The Transformer, predicated on this mechanism, has enabled significant progress in the fields of natural language processing [43,44] and machine vision [45,46,47]. Recently, the self-attention mechanism has gained significant prominence in the field of EEG signal processing, reflecting its growing importance and application. Song et al. [48] proposed a compact convolutional Transformer to encapsulate local and global features in a unified EEG classification framework. This framework utilizes the self-attention module straightforwardly connected to extract the global correlation within the local temporal features. Zhang et al. [49] used the self-attention mechanism and cross-attention mechanisms to achieve interactions between information from different subjects. Li et al. [50] proposed the DABAN model, which leverages a self-attention mechanism to extract features within each domain. This model achieves adversarial learning through the output discrepancy between two classifiers, thereby enhancing the classification performance for target subjects. Lastly, the GAT model [51] utilizes data from different subjects to construct a novel attention-based adaptor, significantly improving the classification performance of the target subjects.

## 3. Methods

### 3.1. Overview

Addressing the cross-subject classification problem within a heterogeneous label space, we select a single subject as the target subject and other subjects as source subjects, thus dividing it into two different but related parts, the source domain $D_{s}$ and the target domain $D_{t}$, with both domains possessing distinct label spaces, $Y_{s}$ and $Y_{t}$. Given the inherent inter-subject variability and label space inconsistency, this study aims to devise an innovative model architecture that effectively tackles these challenges and achieves precise cross-subject classification.

**Figure 2:**
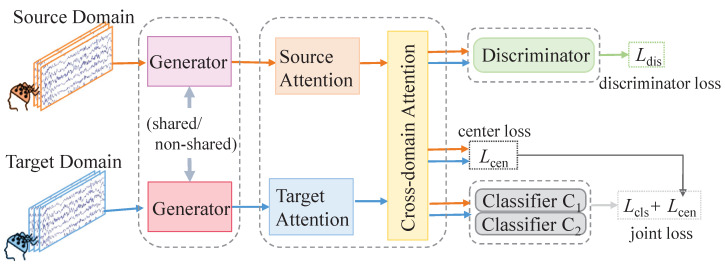
The proposed framework. Blue lines indicate the data flow within the target domain; orange lines depict the data flow originating from the source domain.

To this end, we propose a cross-attention-based class alignment network (CCAN), whose focus lies in deeply exploring the latent features of samples and facilitating the alignment of corresponding class features between the source and target domains through a meticulously designed mechanism, while simultaneously enhancing the discrimination between non-corresponding class features. As shown in Figure 2, this model comprises four pivotal components: the generator, cross-encoder, class discriminator, and classifier. Firstly, the generator module leverages convolutional neural networks (CNNs) to extract preliminary temporal and spatial features from samples in both the source and target domains. These shallow features serve as a foundation for subsequent feature fusion and deep mining. Subsequently, the extracted features are fed into the cross-encoder, which incorporates an attention mechanism to distill highly discriminative deep features. This step ensures that the model can capture salient patterns that are crucial for cross-domain classification. Next, the class discriminator module maps these deep features into a novel feature space. Through a carefully crafted loss function, it achieves two objectives simultaneously: firstly, it brings features from different domains but belonging to corresponding classes closer together, fostering feature alignment; secondly, it separates features of non-corresponding classes, enhancing the inter-class discrimination. This crucial step is paramount to achieving effective cross-subject classification. Finally, the classifier makes the ultimate classification decision based on the optimized features.

The following sections delve into the specifics of each component in detail.

### 3.2. Network Architecture

#### 3.2.1. Generator

EEG signals carry rich information across both temporal and spatial dimensions. To ensure that the integrity of the information in each dimension is preserved during feature extraction, we are inspired by the research of Song et al. [51] and employ two independent pathways for shallow feature extraction. The first pathway focuses on capturing temporal information, utilizing 40 convolutional kernels with a length of *L* to perform temporal convolutions, followed by further processing in the spatial dimension with 40 convolutional kernels of length *C*. Conversely, the second pathway adopts the opposite strategy, initially capturing spatial information before proceeding with temporal convolutions.

Within each pathway, we integrate batch normalization (BN) and an exponential linear unit (ELU) to enhance the expressive power of the combined features. Additionally, to mitigate the risk of overfitting, we implement dropout and average pooling (AvgPool) strategies to improve the generalization ability of the model. Ultimately, the features generated by these two pathways are effectively fused to capture the complex information present in the EEG signals across both the temporal and spatial dimensions.

#### 3.2.2. Cross-Encoder

**Figure 3:**
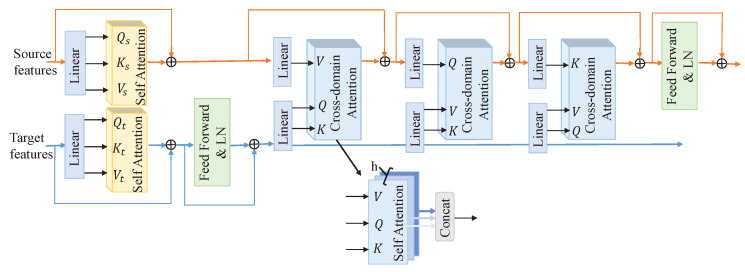
Cross-encoder framework. The blue line represents the target domain data flow, while the orange line signifies the source domain data flow.

MI-EEG signals often span over extended temporal periods. However, the convolutional operations employed in generators are constrained by their limited receptive fields, potentially leading to incomplete feature extraction from EEG signals and particularly struggling to adequately capture the long-term dependencies within the signal’s time series. Given that long-term dependencies are crucial in understanding EEG signals, we observe that the self-attention mechanism has demonstrated remarkable benefits in addressing remote dependency issues and has been widely and successfully applied in various domains, including natural language processing, computer vision, and EEG analysis. Therefore, in this section, we design a cross-encoder based on the attention mechanism, as illustrated in Figure 3, aiming to comprehensively capture the complex features and long-term dependencies present in MI-EEG signals.

In the first step, we independently apply self-attention operations to the source and target domains to learn the global dependency relationships within their respective domain features. The output features from the generator are sliced along the feature dimension, resulting in *h* segments, each with a dimension of t×d, referred to as “tokens”, where each token represents a small feature slice. Subsequently, through linear transformations, these tokens are converted into three identical copies, serving as the query (*Q*), key (*K*), and value (*V*), respectively. To prevent gradient vanishing issues during subsequent operations, we introduce a scaling factor *k*,

(1) $$\mathrm{Attention}\left(Q,K,V\right)=\mathrm{Softmax}\left(\frac{QK^{T}}{\sqrt{k}}\right)V.$$

To enhance the feature representation diversity, we employ a multi-head strategy. The generator’s output features are split into *h* segments, and each segment independently undergoes self-attention operations. Subsequently, the parallel results from these operations are merged and output. This process is represented as follows:

(2) $$\mathrm{MHA}\left(Q,K,V\right)=\left[{\mathrm{head}}_{0},{\mathrm{head}}_{1},\ldots ,{\mathrm{head}}_{h-1}\right],$$

(3) $${\mathrm{head}}_{l}=\mathrm{Attention}\left(Q_{l},K_{l},V_{l}\right).$$

Then, we enhance the self-attention mechanism of the target domain by integrating a feed-forward network coupled with layer normalization.

In the second step, building upon the initial self-attention phase, we apply a multi-head cross-domain attention mechanism to the source domain. During this process, the source domain sequentially provides *V*, *Q*, and *K*, while the target domain flexibly adapts by sequentially presenting combinations of *Q* with *K*, *V* with *K*, and *V* with *Q*. In this way, each attention operation integrates the three copies from both the source and target domains, aiming to enable the source features to learn from the target features, thereby further reducing the discrepancy between the two domains. Following this step, the source features undergo processing by a feed-forward network and are subjected to layer normalization to ensure the stability of the data distribution.

#### 3.2.3. Class Discriminator

Given the inherent variability among different subjects, even within the same category, features from different domains exhibit a certain degree of variation. Merely relying on cross-encoders to reduce the disparities between the domains is insufficient. To achieve the more comprehensive and precise alignment of features belonging to the corresponding category across the source and target domains, we devise a class discriminator. This discriminator initially employs layer normalization (LN), followed by a fully connected layer with *r* neurons.

Existing discriminator approaches primarily focus on minimizing the marginal probability distribution discrepancy between the source and target domain features, often neglecting the crucial impact of changes in the conditional probability distributions under given classification conditions on classification tasks. To address this issue, we propose a novel loss function specifically designed to encourage the convergence of features within corresponding classes while promoting divergence among features from non-corresponding classes. The formula is detailed as follows:

(4) $$L_{\mathrm{dis}}=\frac{L_{\mathrm{corr}}}{L_{\mathrm{ncorr}}},$$

where $L_{\mathrm{corr}}$ and $L_{\mathrm{ncorr}}$ are defined, respectively, as follows:

(5) $$L_{\mathrm{corr}}=\frac{1}{\tilde{M}}\sum_{m=1}^{\tilde{M}}\rho \left({\bar{F}}_{m}^{s},{\bar{F}}_{m}^{t}\right),$$

(6) $$L_{\mathrm{ncorr}}=\frac{1}{\tilde{M}\left(\tilde{M}-1\right)}\sum_{\begin{array}{c} m=1\\ m\neq m^{\prime } \end{array}}^{\tilde{M}}\sum_{m^{\prime }=1}^{\tilde{M}}\rho \left({\bar{F}}_{m}^{s},{\bar{F}}_{m^{\prime }}^{t}\right),$$

where $\tilde{M}$ represents the number of corresponding classes present in the current batch. The function ρ(·,·) is utilized to measure the similarity, with the L2-norm employed as the standard for the distance metric. For a given class label *m*, ${\bar{F}}_{m}^{s}$ and ${\bar{F}}_{m}^{t}$ represent the average features of the corresponding classes of samples in the source and target domains, respectively. Furthermore, for two non-corresponding class labels *m* and $m^{\prime }$, ${\bar{F}}_{m}^{s}$ and ${\bar{F}}_{m^{\prime }}^{t}$ represent the average features of samples belonging to non-corresponding classes from the source and target domains, respectively.

The loss function $L_{\mathrm{corr}}$ quantifies the feature discrepancies between samples of the corresponding class across the source and target domains. Conversely, $L_{\mathrm{ncorr}}$ measures the feature discrepancies of samples from non-corresponding classes within these domains. Therefore, by minimizing the loss function $L_{\mathrm{dis}}$, we strive to minimize the feature distance for samples of the corresponding class while maximizing it for those from non-corresponding classes, thereby promoting feature homogeneity within the classes and heterogeneity between them.

#### 3.2.4. Classifier

We devise two parallel classifiers that operate in concert to optimize the decision-making process. Initially, we randomly initialize these classifiers with distinct sets of initial weights and biases, a strategy aimed at introducing perturbations that subsequently enhance the diversity and robustness of the network. Subsequently, both classifiers share the following classification loss function, which ensures the consistency of their classification performance:

(7) $$L_{\mathrm{cls}}=-\frac{1}{B_{n}}\sum_{i=1}^{N}\sum_{m=1}^{M}y_{im}\log\left({\hat{y}}_{im}\right),$$

where *M* denotes the total number of EEG categories, and $B_{n}$ represents the batch size. The term $y_{im}$ corresponds to the *m*-th value in the one-hot-encoded vector for the *i*-th sample. Meanwhile, $\hat{y_{im}}$ denotes the predicted probability that the *i*-th sample is classified under the *m*-th category.

During testing, the final prediction is obtained by averaging the outputs of the two classifiers. This method mitigates the potential error or bias inherent in a single classifier, thereby enhancing the overall network’s generalization capabilities and stability.

### 3.3. Training Procedure

Our network is trained in two steps, using the ADAM optimizer to ensure consistency between the two stages of the stepwise training strategy. In the first step, we train the generator, cross-encoder, and class discriminator. In the second step, we train the generator, cross-encoder, and classifier.

To further reduce the intra-class differences, we align the central features of the source domain and target domain, introducing the following loss function:

(8) $$L_{\mathrm{cen}}=\sum_{m}\left(\frac{1}{N_{m}}\sum_{i=1}^{N_{m}}∥f_{mi}-{\bar{f}}_{c}^{t}{∥}^{2}\right),$$

where $f_{mi}$ represents the feature of the *i*-th sample within the *m*-th category. These features are extracted from the samples in both the source and target domains. $N_{m}$ denotes the total number of samples belonging to the *m*-th category. The term ${\bar{f}}_{m}^{t}$ represents the central feature of samples categorized under the *m*-th category in the target domain. It is worth noting that a batch of training data includes samples from both the source and target domains. Consequently, $f_{mi}$ may refer to either a feature from a source domain sample or a feature from a target domain sample.

Subsequently, we combine $L_{\mathrm{cls}}$ with $L_{\mathrm{cen}}$ to derive the joint loss function, expressed as

(9) $$L_{\mathrm{joint}}=L_{\mathrm{cls}}+w_{\mathrm{cen}}L_{\mathrm{cen}},$$

where $w_{\mathrm{cen}}$ serves as a hyperparameter that modulates the influence of $L_{\mathrm{cls}}$ and $L_{\mathrm{cen}}$.

## 4. Experiments

### 4.1. Dataset and Data Preprocessing

We evaluate our approach using two publicly available EEG datasets from the BCI Competition IV, known as Dataset 2a [52] and Dataset 1 [53].

#### 4.1.1. Dataset 2a of BCI Competition IV

This dataset encompasses recordings from 9 healthy subjects, utilizing 22 EEG channels and 3 EOG channels, with a sampling rate of 250 Hz. Each subject was asked to perform four MI tasks: left hand, right hand, feet, and tongue. Each subject participated in 2 sessions, with each session consisting of 288 trials, equally divided among the four MI tasks (i.e., 72 trials per task). In our experimental setup, the EOG data were disregarded, and only the EEG signals spanning [2, 6] s of each trial were extracted for analysis. Subsequently, a 3-order Butterworth bandpass filter was employed to restrict the frequency range of the EEG data to [4, 38] Hz, effectively eliminating artifacts and noise.

#### 4.1.2. Dataset 1 of BCI Competition IV

This dataset contains EEG recordings from 59 channels for 7 healthy subjects, with a sampling rate of 100 Hz. Each subject participated in 3 sessions, but, for this study, our focus is solely on the data from the training session. In this session, each subject contributed 200 samples, with the sample categories being selected from one of the combinations of “left hand, right hand” and “left hand, foot” and labeled as +1 and −1 in the file. In our experiment, only the 4 s EEG signals from each MI trial were extracted as our experimental data. Subsequently, a 3-order Butterworth bandpass filter was applied to restrict the frequency range of the EEG data to [4, 38] Hz, effectively removing artifacts and noise.

We use z-score normalization to preprocess the EEG data, as shown below:

(10) $${\tilde{x}}_{i,c,t}=\frac{x_{i,c,t}-{\bar{x}}_{c,t}}{\sigma_{c,t}},$$

where $x_{i,c,t}$ represents the original value of the *i*-th filtered sample at position (c,t), ${\tilde{x}}_{i,c,t}$ is its standardized value, ${\bar{x}}_{c,t}$ is the mean value of the samples at position (c,t), and $\sigma_{c,t}$ denotes the standard deviation of the samples at the same position.

### 4.2. Experimental Settings

Throughout the experimental phase, our computations were performed using Pytorch on a server platform, which was outfitted with both an Intel(R) Xeon(R) Silver 4110 CPU and NVIDIA Tesla P100-PCIE GPU. The model was trained utilizing the ADAM optimizer, with the learning rate set at 0.0002. The exponential decay rates for the first- and second-order moment estimates, denoted as $\beta_{1}$ and $\beta_{2}$, were set to 0.5 and 0.999, respectively. When dealing with Dataset 2a or Dataset 1, we set the value of *L* to 25 or 10, respectively. Specifically, in the first four scenarios, the generator for the source and target domains was designed to be shared; meanwhile, in scenario 5, the generator was not shared. Additionally, the batch size was configured to 64.

Consistent with the existing literature, we employed the classification accuracy as a metric to assess the performance of the MI-EEG model. These values were derived from the M×M confusion matrix $\left(a_{ij}\right)$, where $a_{ij}$ represents the number of test samples that are truly labeled as class *i* but are predicted by the model to be class *j*.

The accuracy is defined as

(11) $$Acc=\frac{\sum_{i=1}^{M}a_{ii}}{N},$$

where $N=\sum_{i=1}^{M}\sum_{j=1}^{M}a_{ij}$ is the total number of test samples.

### 4.3. DA Scenarios

We adopted five DA scenarios from [28], as shown in Table 1.

For the first four scenarios, the source and target domains share the same feature space but differ in their label spaces. To achieve this, we selected one subject from Dataset 2a as the target subject, while the remaining subjects served as source subjects. For each scenario, we chose three examples, which are presented in Table 1. Scenario 1: The source and target domains share one common label and each possesses one distinct label, resulting in 24 possible binary classification problems. Specifically, we use the notation 1,2→1,3 to represent the selection of data with labels 1 and 2 from the source domain and labels 1 and 3 from the target domain. The labels before and after the symbol → correspond in order. Since the case of 1,2→1,3 is equivalent to that of 2,1→3,1, we fix the labels in front of the arrows in sequential order. This applies to all other scenarios as well. Scenario 2: The two domains share two common labels and each possesses one distinct label, yielding 12 possible ternary classification problems. Scenario 3: There are 6 possible binary classification problems where the label spaces of the two domains are completely distinct. Scenario 4: We modify the configuration of scenario 2 to obtain 12 types of ternary classification problems with completely different labels. In other words, we swap the positions of the first two labels in the target domain to ensure that the matched labels between the two domains are completely different.

For the last scenario, the source and target domains differ in both the feature spaces and their label spaces. We select seven subjects from Dataset 1 as source subjects and one subject from Dataset 2a as the target subject. This scenario involves a binary classification problem, using data labeled as 3 and 4 in the target domain and data labeled as +1 and −1 in the source domain. There is 1 possible binary classification problem.

### 4.4. Baseline Comparison

**Figure 4:**
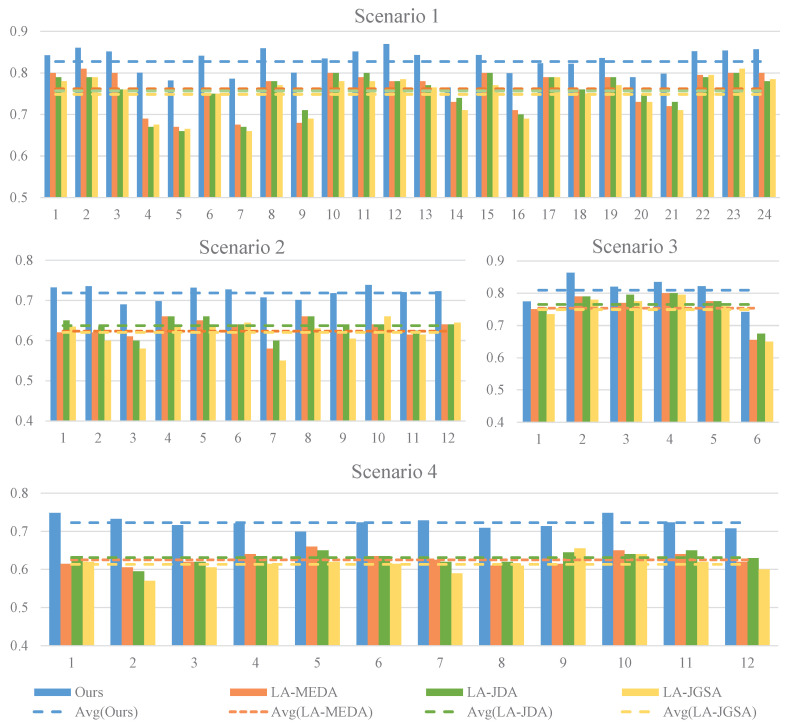
Comparison results with existing methods across the first four scenarios. The horizontal axis represents the serial number of classification possibilities in each scenario, while the vertical axis indicates the average accuracy of the nine target subjects.

**Figure 5:**
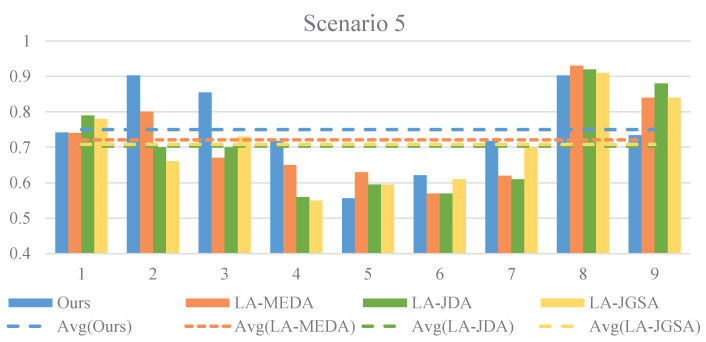
Comparison results with existing methods in Scenario 5. The horizontal axis represents the serial number of subjects in the target domain, while the vertical axis represents their accuracy.

We evaluate our algorithm across various scenarios on two datasets, as shown in Figure 4 and Figure 5. To validate the effectiveness of our approach, we conduct a comparative analysis with three machine learning methods [28], namely LA-MEDA, LA-JDA, and LA-JGSA, which are renowned for their outstanding classification performance. These methods integrate LA data preprocessing techniques with domain adaptation methods, exhibiting impressive performance. Our comparative results reveal that, in the first four scenarios, our method outperforms all other methods across all classification combinations within each scenario. In scenario 5, while our method does not achieve optimal results for some specific subjects, it still surpasses the other three methods in terms of its average performance. In summary, based on the overall average results, our method demonstrates superior performance across all five scenarios, providing compelling evidence of its significant advantages and effectiveness in addressing heterogeneous data migration problems.

### 4.5. Ablation Experiment

#### 4.5.1. Ancillary Effects of Source Domains

To evaluate the rationality and effectiveness of our approach in leveraging source domain data, we selected a classification task from each of the five scenarios as the baseline and designed two comparative experiments. They were designed to delve into the contributions of the source data in cross-subject classification problems and to demonstrate the superiority of our proposed framework in addressing these challenges. In experiment 1, we maintained the original framework but completely replaced the source domain data, originally used for auxiliary training, with target domain data. In experiment 2, we structurally modified the framework by removing all components that directly utilized source domain data, retaining only the generator, the target domain attention mechanism within the cross-encoder, and the classifiers.

As shown in Table 2, the average accuracy of the baseline experiments surpasses that of the two comparative experiments without utilizing source data in the first four scenarios. This finding underscores the positive and pivotal role that the source data play in enhancing the performance of target domain models within the context of heterogeneous label spaces, further validating the rationality and effectiveness of our approach in leveraging source data efficiently. Delving deeper into the analysis, intriguing phenomena emerge in the binary classification tasks. In scenario 1, the classification performance of the baseline model significantly surpasses that in both comparative experiments by approximately 3%, highlighting the substantial positive impact of the source data in this scenario. Conversely, in scenario 3, this advantage diminishes markedly, as the performance gap between the baseline model and comparative experiments narrows to less than 1%, indicating that the positive effect of the source data is mitigated by the completely heterogeneous nature of the categories. Turning to ternary classification tasks, we observe that the classification performance of the baseline models in scenarios 2 and 4 exhibits minimal differences and both significantly outperform the comparative experiments without source data. This result underscores the capability of our method to harness the positive influence of source data effectively, even in the face of more complex classification tasks, particularly when dealing with completely heterogeneous categories, thereby ensuring stable and superior classification performance.

However, it is noteworthy that an unexpected reversal occurs in scenario 5, where the average accuracy of the baseline experiment falls below that of the experiment without source data. This anomalous phenomenon reveals that when there exist discrepancies in the feature spaces between the source and target domains, the source data can adversely affect the performance of target domain models, subsequently leading to a decline in classification effectiveness. This issue will be a crucial focus of our future research endeavors.

#### 4.5.2. Effect of Cross-Encoder

**Figure 6:**
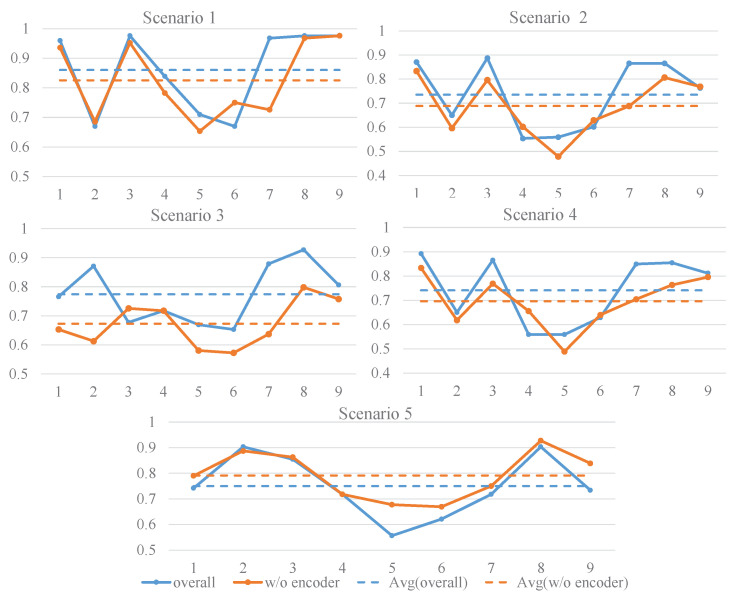
Comparison of effects with and without cross-encoder. The horizontal axis depicts the number of subjects, and the vertical axis continues to represent the classification accuracy. Scenario 1: 1,2→1,4. Scenario 2: 1,2,4→1,2,3. Scenario 3: 1,2→3,4. Scenario 4: 1,2→3,4. Scenario 5: +,−→3,4.

We evaluated the necessity of the cross-encoder within our overall framework by removing it and retraining the models. We then randomly selected one configuration from each scenario as a representative and present the performance results in Figure 6.

As shown in Figure 6, models equipped with the cross-encoder exhibit higher average accuracy compared to those without it across scenarios 1–4. This observation aligns with our initial expectations and underscores the cross-encoder’s ability to capture more salient features of the target domain, thereby enhancing the classification performance on target domain data. However, in scenario 5, an inverse trend emerges, with the model without the cross-encoder demonstrating superior performance.

After a meticulous comparison of the differences between this scenario and the preceding four, we found that the fifth scenario involves heterogeneous feature spaces. We speculate that, in such an environment, although the cross-encoder is designed to promote interaction and fusion between features, the interaction process may not be as smooth and effective as expected due to the significant differences and uneven distribution of nonlinear relationships between the features. Consequently, the cross-encoder may not fully demonstrate its advantages in this scenario and could introduce noise or interference due to inappropriate feature fusion methods, ultimately affecting the model’s classification performance.

#### 4.5.3. Effect of Class Discriminator

To delve into the role of the class discriminator within our proposed framework, we removed this component and conducted experiments on a binary classification task (1,2→1,4) and a ternary classification task (1,2,4→2,1,3). Additionally, we varied the number of output neurons *r* in the class discriminator, setting it to 2, 3, 4, and 5, respectively, to comprehensively evaluate its impact on the model performance.

The results presented in Table 3 demonstrate that models incorporating a class discriminator consistently achieve higher average classification accuracy compared to those without a class discriminator across two distinct classification tasks. This finding validates the effectiveness of the class discriminator in enhancing the feature discrimination capabilities. Additionally, we observe that *r* does not exhibit a clear optimal value or consistent regularity across the two classification tasks. We also conducted experiments in scenario 2 (1,2,3→1,2,4) and scenario 3 (1,2→3,4), yet the optimal value remains elusive. This suggests that the choice of *r* may not be a decisive factor in the final model’s performance and should be considered comprehensively, taking into account the auxiliary role of the class discriminator architecture.

### 4.6. Time Complexity Analysis

This section investigates the time complexity of the entire algorithm across five scenarios. Since all classification instances within the same scenario are based on an identical sample size, we selected one experiment from each scenario to evaluate the model’s training duration and the testing time per sample. The hardware used for these experiments included an Intel(R) Xeon(R) Silver 4110 CPU and an NVIDIA Tesla P100-PCIE GPU. The experimental results are summarized in Table 4. The training time is defined as the time consumed for the training of a classification model for one target subject over 500 epochs. The testing time is the average time per sample after 100 repeated tests. Scenarios 1 and 3 have identical sample sizes, resulting in comparable training times; similarly, scenarios 2 and 4 share the same sample sizes, leading to comparable training times. Since all scenarios employ the same model framework, the testing time per sample is almost identical across the different scenarios.

### 4.7. Visualization

**Figure 7:**
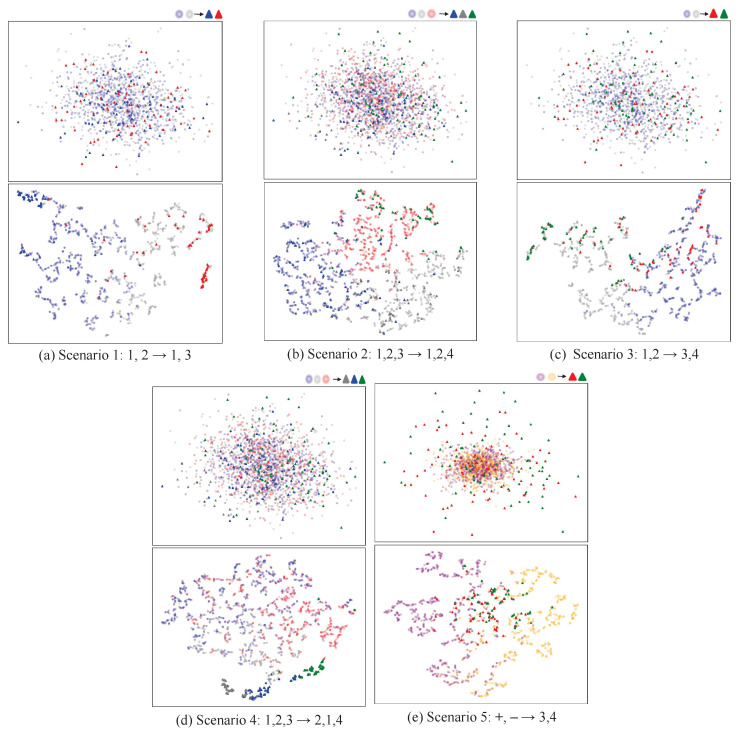
Original feature distributions (**top**) and feature distributions extracted by our framework (**bottom**). Blue: left hand, gray:right hand, green: tongue, red: feet.

To evaluate the performance of our framework in feature extraction, we utilized the t-SNE algorithm for feature visualization. We randomly selected a classification task from each scenario and present the results in Figure 7.

Throughout the feature visualization, we consistently targeted subject A01 from Dataset 2a to ensure the uniformity and comparability of our analysis. In Figure 7, the top section showcases the original feature distribution states of the five classification tasks after preprocessing, while the bottom section illustrates the feature distribution extracted by our framework.

Firstly, we compare the changes in the feature distributions before and after extraction. Initially, the feature distributions in the five classification tasks are disordered, with significant overlap among features from different classes. However, after applying our proposed framework, the features within each class become as clustered as possible, while features from different classes are as separated as possible.

Secondly, we contrast the original feature distributions across the five scenarios. Compared to scenario 5, the first four scenarios exhibit a more uniform trend in the feature distributions between the source and target domains, with similar distributions of both circles and triangles. This indicates good consistency in the feature space between the source and target domains in these scenarios. In contrast, the original feature distribution of scenario 5 significantly differs from that of the other scenarios, with a notably larger distribution range of target domain features compared to the source domain, directly reflecting the distinct feature spaces between the source and target domains in this scenario. This heterogeneity complicates the interaction between the features of the source and target domains, posing a challenge to the feature fusion of the cross-encoder for the classification task in scenario 5 and preventing it from achieving the expected performance level.

Lastly, we compare the distribution of the extracted features across the five scenarios, highlighting the varied performance of our method.

Binary Classification Task: Scenario 1: There is clear separation between the blue and red triangles from the target domain, and the blue and gray circles from the source domain also have distinct boundaries, clustering closely around their respective class samples. This indicates that our method can extract discriminative features that facilitate the classification task when the source and target domains share one label. Under this condition, our method also effectively aligns the source domain samples with those of the target domain in the feature space, enabling successful knowledge transfer. Furthermore, our method aggregates samples of the same class while separating those of different classes. These feature achievements are reflected in the final classification performance: as depicted in Figure 4, scenario 1 achieves significantly higher average accuracy compared to the other scenarios. Scenario 3: The source domain samples are well distributed around their corresponding class samples in the target domain, indicating the successful alignment of the source domain features to the target domain features based on the prescribed classes. However, some overlap exists between the green and red triangles from the target domain, resulting in lower average accuracy compared to scenario 1 (as shown in Figure 4). This suggests that the impact of source domain samples with completely heterogeneous labels on the target domain is reduced (as shown in Table 2). Scenario 5: The distribution areas of red triangles and green triangles happen to lie at the edges of the boundary dividing the purple circles and yellow circles. This phenomenon underscores the discrepancy in the distribution ranges between the source domain samples and target domain samples, corroborating the statement in Section 4.5.1 that, in scenario 5, the source domain data adversely affect the performance of the target domain model. We hypothesize that this may be attributed to the utilization of non-shared generators between the source domain and the target domain. This mechanism of non-shared generators potentially induces significant feature discrepancies, leading to an adverse effect on the target domain model’s performance when the source domain data are used to assist in its training. It is noteworthy that there exists significant overlap between the red triangles and green triangles, making it nearly impossible to delineate a clear boundary, which directly contributes to the decline in model accuracy (as shown in Table 2). This result indicates that, under the circumstances in which both the original feature space and labels are heterogeneous, it is more challenging for our method to exert a positive effect. This represents a key focus of our future research.

Ternary Classification Task: Scenarios 2 and 4: The distribution boundaries of the three colored triangles from the target domain exhibit similar patterns: some overlap between blue and gray triangles, with a clearer boundary for green triangles. This observation is consistent with the result that the average accuracy of these two scenarios is comparable (as shown in Figure 4). However, the source domain distributions in the two scenarios differ notably: in scenario 2, where the source and target domains share two labels, the source domain distribution aligns well with that of the target domain. In contrast, scenario 4 features completely heterogeneous labels between the source and target domains, leading to a non-class-specific distribution and the non-alignment of the source domain to the target domain. Nonetheless, the accuracy in scenario 4 remains comparable to that of scenario 2, suggesting that, even in the presence of completely heterogeneous classes, our method can harness the source domain positively, achieving stable classification performance. This underscores the effectiveness and robustness of our approach in addressing heterogeneous space issues.

In summary, the performance variations of our method across different scenarios reveal the critical influence of factors such as the consistency of the feature spaces between the source and target domains, the extent of label space overlap, and the experimental settings on the effectiveness of knowledge transfer from the source domain to the target domain.

## 5. Discussion and Conclusions

In the realm of brain electrophysiological decoding, most current DA strategies focus on homogeneous transfer learning, where the source and target domains share identical feature spaces and label spaces. However, this assumption often does not hold true in real-world applications. To address this, this paper innovatively proposes a deep learning-based network architecture.

(1) Our method effectively integrates known information from the source domain with limited data from the target domain, facilitating transfer learning in a heterogeneous space. This approach significantly enhances the efficiency and effectiveness of cross-subject information transfer.

(2) The designed cross-encoder facilitates the interaction and fusion of source and target domain features within the same feature space, thereby improving the classification performance for target domain data.

We are acutely aware of the limitations inherent in our current approach, particularly when dealing with completely heterogeneous feature spaces, as exemplified in scenario 5. The EEG data from distinct feature spaces necessitate the employment of generators with varying sizes. However, the necessity to maintain feature dimensionality consistency often leads to the selection of suboptimal generator sizes, undeniably increasing the risk of feature information loss. Consequently, our future research endeavors will be directed towards the development of more flexible feature extraction strategies, with the aim of comprehensively addressing the challenges posed by heterogeneous feature spaces.

## Figures and Tables

**Table 1:** Description of the scenarios.

Scenario	No. of Classes	Features	Labels	No. of Classifications	Examples (Source → Target)
					1,2→1,3
1	2	same	partially different	24	2,3→2,4
					2,4→2,1
					1,2,3→1,2,4
2	3	same	partially different	12	1,2,3→1,4,3
					1,2,4→1,2,3
					1,2→3,4
3	2	same	completely different	6	1,3→2,4
					3,4→1,2
					1,2,3→2,1,4
4	3	same	completely different	12	1,2,4→2,1,3
					1,3,4→3,1,2
5	2	different	completely different	1	+,−→3,4

**Table 2:** Accuracy (%) of the two variants without the source domain. A01–09 are subject IDs in Dataset 2a. The symbols S, T, and E&D represent source data, target data, and the cross-encoder and discriminator, respectively. ✓ indicates the use of that data or module. Scenario 1: 1,2→1,3. Scenario 2: 1,2,3→1,2,4. Scenario 3: 1,2→3,4. Scenario 4: 1,2,3→2,1,4. Scenario 5: +,−→3,4.

Scenario	Condition			A01	A02	A03	A04	A05	A06	A07	A08	A09	Avg.
	S	T	E&D										
	✓	✓	✓	**96.77**	87.10	85.48	**76.61**	**62.10**	75.00	95.16	**92.74**	**87.10**	**84.23**
1		✓	✓	94.36	**88.71**	80.65	75.00	59.68	68.55	**97.58**	82.26	82.29	81.45
		✓		87.10	82.26	**86.29**	73.39	61.29	**76.61**	96.77	86.29	83.87	81.54
	✓	✓	✓	**89.25**	56.99	**93.55**	**68.82**	**54.30**	**54.84**	**77.42**	**93.55**	**70.43**	**73.24**
2		✓	✓	83.33	**51.61**	87.63	59.14	55.38	54.84	82.80	90.32	71.50	70.73
		✓		80.65	44.62	87.63	52.15	53.22	55.38	75.27	87.10	76.88	68.10
	✓	✓	✓	**76.61**	87.10	67.74	71.77	**66.94**	65.32	**87.90**	**92.74**	80.65	**77.42**
3		✓	✓	**76.61**	87.90	**75.81**	66.94	**66.94**	**66.13**	87.10	89.52	75.81	76.97
		✓		74.19	**89.52**	77.42	**79.03**	56.45	63.71	82.26	90.32	**83.07**	77.33
	✓	✓	✓	**90.32**	**51.61**	**93.55**	**67.20**	50.54	**62.37**	**85.48**	**94.62**	**77.96**	**74.85**
4		✓	✓	83.33	**51.61**	87.63	59.14	**55.38**	54.84	82.80	90.32	71.50	70.73
				80.65	44.62	87.63	52.15	53.22	55.38	75.27	87.10	76.88	68.10
	✓	✓	✓	74.19	**90.32**	**85.48**	71.77	55.65	62.10	81.77	**90.32**	73.39	75.00
5		✓	✓	**76.61**	87.90	75.81	66.94	**66.94**	**66.13**	**87.10**	89.52	75.81	76.97
		✓		74.19	89.52	77.42	**79.03**	56.45	63.71	82.26	**90.32**	**83.07**	**77.33**

**Table 3:** Accuracy (%) with and without class discriminator. When r=0, it indicates the absence of the class discriminator. A01–09 are subject IDs in Dataset 2a. Scenario 1: 1,2→1,4, Scenario 4: 1,2,4→2,1,3.

Scenario	*r*	A01	A02	A03	A04	A05	A06	A07	A08	A09	Avg.
	2	97.58	66.13	98.39	84.68	70.16	68.55	95.96	96.77	97.58	86.20
	3	95.97	66.94	99.19	83.07	69.36	68.55	97.58	97.58	97.58	86.20
1	4	96.77	69.35	97.58	83.87	70.96	66.93	96.77	99.19	96.77	86.47
	5	95.97	66.94	97.58	83.87	70.97	66.94	96.77	97.58	97.58	86.02
	0	95.97	66.93	98.39	83.07	70.16	65.32	95.97	98.39	98.39	85.84
	2	90.32	68.28	87.63	57.53	55.91	61.29	84.95	84.95	79.03	74.43
	3	90.32	66.13	87.10	57.53	51.07	62.90	90.32	85.48	76.88	74.19
4	4	89.25	68.28	87.63	56.99	55.91	60.21	86.02	83.87	75.27	73.72
	5	89.25	65.05	86.56	55.91	55.91	62.90	84.95	85.48	81.18	74.13
	0	89.25	63.44	86.56	57.53	54.84	59.14	84.41	83.33	77.42	72.88

**Table 4:** Training and inference times across five scenarios.

Scenario	Training Time (min)	Testing Time (ms)
1	23	5.64
2	36	5.58
3	23	6.04
4	37	5.60
5	31	5.79

## Data Availability

The data that support the findings of this study are openly available at the following URL/DOI: https://www.bbci.de/competition/iv/ (accessed on 31 October 2024).

## References

[1] Lance B.J., Kerick S.E., Ries A.J., Oie K.S., McDowell K. (2012). Brain-computer interface technologies in the coming decades. Proc. IEEE.

[2] Wolpaw J.R., Birbaumer N., McFarl D.J., Pfurtscheller G., Vaughan T.M. (2002). Brain-computer interfaces for communication and control. Clin. Neurophysiol..

[3] Liu Y., Ding Y., Li C., Cheng J., Song R.C., Wan F., Chen X. (2020). Multi-channel EEG-based emotion recognition via a multi-level features guided capsule network. Comput. Biol. Med..

[4] Islam M.R., Islam M.M., Rahman M.M., Mondal C., Singha S.K., Ahmad M., Awal A., Islam M.S., Moni M.A. (2021). EEG channel correlation based model for emotion recognition. Comput. Biol. Med..

[5] Wen Y.Z., Zhang Y.J., Wen L., Cao H.J., Ai G.P., Gu M.H., Wang P.J., Chen H.L. (2022). A 65 nm/0.448 mW EEG processor with parallel architecture SVM and lifting wavelet transform for high-performance and low-power epilepsy detection. Comput. Biol. Med..

[6] Oliva J.T., Rosa J. (2021). Binary and multiclass classifiers based on multitaper spectral features for epilepsy detection. Biomed. Signal Process. Control.

[7] Zhang Y.J., Ma J.F., Zhang C., Chang R.S. (2021). Electrophysiological frequency domain analysis of driver passive fatigue under automated driving conditions. Sci. Rep..

[8] Min J.L., Xiong C., Zhang Y.G., Cai M. (2021). Driver fatigue detection based on prefrontal EEG using multi-entropy measures and hybrid model. Biomed. Signal Process. Control.

[9] Kim K.-T., Carlson T., Lee S.-W. Design of a robotic wheelchair with a motor imagery based brain-computer interface. Proceedings of the 2013 International Winter Workshop on Brain-Computer Interface, BCI.

[10] Krusienski D.J., Shih J.J. Spectral components of the P300 speller response in and adjacent to the hippocampus. Proceedings of the 2012 IEEE International Conference on Systems, Man, and Cybernetics, SMC.

[11] Shi M.H., Zhou C.L., Xie J., Li S.Z., Hong Q.Y., Jiang M., Chao F., Ren W.F., Liu X.Q., Zhou D.J. (2018). Electroencephalogram-based brain-computer interface for the Chinese spelling system: A survey. Front. Inf. Technol. Electron. Eng..

[12] Sadeghi S., Maleki A. (2020). Character encoding based on occurrence probability enhances the performance of SSVEP-based BCI spellers. Biomed. Signal Process. Control.

[13] Chen X.G., Zhao B., Wang Y.J., Gao X.R. (2019). Combination of high frequency SSVEP-based BCI and computer vision for controlling a robotic arm. J. Neural Eng..

[14] Chen X.G., Zhao B., Wang Y.J., Gao X.R. (2020). Combination of augmented reality based brain-computer interface and computer vision for high-level control of a robotic arm. IEEE Trans. Neural Syst. Rehabil. Eng..

[15] Grosse-Wentrup M., Buss M. (2008). Multiclass common spatial patterns and information theoretic feature extraction. IEEE. Trans. Biomed. Eng..

[16] Ang K.K., Chin Z.Y., Zhang H., Guan C. Filter bank common spatial pattern (FBCSP) in brain-computer interface. Proceedings of the 2008 IEEE International Joint Conference on Neural Networks (IEEE World Congress on Computational Intelligence).

[17] Kant P., Laskar S.H., Hazarika J., Mahamune R. (2020). CWT based transfer learning for motor imagery classification for brain computer interfaces. J. Neurosci. Methods.

[18] Bhattacharyya A., Singh L., Pachori R.B. (2018). Fourier–Bessel series expansion based empirical wavelet transform for analysis of non-stationary signals. Digit. Signal Process..

[19] Bhattacharyya A., Pachori R.B. (2017). A multivariate approach for patient-specific EEG seizure detection using empirical wavelet transform. IEEE Trans. Biomed. Eng..

[20] Chen C.Y., Wu C.W., Lin C.T., Chen S.A. A novel classification method for motor imagery based on brain-computer interface. Proceedings of the 2014 International Joint Conference on Neural Networks (IJCNN).

[21] Fraiwan L., Lweesy K., Khasawneh N., Wenz H., Dickhaus H. (2012). Automated sleep stage identification system based on time–frequency analysis of a single EEG channel and random forest classifier. Comput. Methods Programs Biomed..

[22] Bishop C.M. (2006). Pattern Recognition and Machine Learning.

[23] Kousarrizi M.R.N., Ghanbari A.A., Teshnehlab M., Shorehdeli M.A., Gharaviri A. Feature extraction and classification of EEG signals using wavelet transform, SVM and artificial neural networks for brain computer interfaces. Proceedings of the 2009 International Joint Conference on Bioinformatics, Systems Biology and Intelligent Computing (IJCBS).

[24] Lawhern V.J., Solon A.J., Waytowich N.R., Gordon S.M., Hung C.P., Lance B.J. (2018). EEGNet: A compact convolutional neural network for EEG-based brain–computer interfaces. J. Neural Eng..

[25] Schirrmeister R.T., Springenberg J.T., Fiederer L.D.J., Glasstetter M., Eggensperger K., Tangermann M., Hutter F., Burgard W., Ball T. (2017). Deep learning with convolutional neural networks for EEG decoding and visualization. Hum. Brain Mapp..

[26] Pan S.J., Yang Q. (2010). A survey on transfer learning. IEEE Trans. Knowl. Data Eng..

[27] He H., Wu D. (2020). Transfer learning for brain–computer interfaces: A Euclidean space data alignment approach. IEEE Trans. Biomed. Eng..

[28] He H., Wu D. (2020). Different Set Domain Adaptation for Brain-Computer Interfaces: A Label Alignment Approach. IEEE Trans. Neural Syst. Rehabil. Eng..

[29] Wu D., Lawhern V.J., Hairston W.D., Lance B.J. (2016). Switching EEG Headsets Made Easy: Reducing Offline Calibration Effort Using Active Weighted Adaptation Regularization. IEEE Trans. Neural Syst. Rehabil. Eng..

[30] Wu H., Xie Q., Yu Z., Zhang J., Liu S., Long J. (2024). Unsupervised heterogeneous domain adaptation for EEG classification. J. Neural Eng..

[31] Busto P.P., Gall J. Open set domain adaptation. Proceedings of the IEEE International Conference on Computer Vision (ICCV).

[32] Saito K., Yamamoto S., Ushiku Y., Harada T. Open set domain adaptation by backpropagation. Proceedings of the European Conference on Computer Vision.

[33] You K., Long M., Cao Z., Wang J., Jordan M.I. Universal domain adaptation. Proceedings of the IEEE/CVF Conference on Computer Vision and Pattern Recognition (CVPR).

[34] Jin Y.M., Luo Y.D., Zheng W.L., Lu B.L. EEG-Based emotion recognition using domain adaptation network. Proceedings of the 2017 International Conference on Orange Technologies, ICOT.

[35] Hang W.L., Feng W., Du R.Y., Liang S., Chen Y., Wang Q., Liu X.J. (2019). Cross-subject EEG signal recognition using deep domain adaptation network. IEEE Access.

[36] Chen P.Y., Gao Z.K., Yin M.M., Wu J.L., Ma K., Grebogi C. (2021). Multiattention adaptation network for motor imagery recognition. IEEE Trans. Syst. Man Cybern. Syst..

[37] Hong X.L., Zheng Q.Q., Liu L.Y., Chen P.Y., Ma K., Gao Z.K., Zheng Y.F. (2021). Dynamic joint domain adaptation network for motor imagery classification. IEEE Trans. Neural Syst. Rehabil. Eng..

[38] Zhao H., Zheng Q.Q., Ma K., Li H.Q., Zheng Y.F. (2020). Deep representation based domain adaptation for nonstationary EEG classification. IEEE Trans. Neural Netw. Learn. Syst..

[39] Raza H., Cecotti H., Li Y.H. (2016). Girijesh Prasad, Adaptive learning with covariate shift-detection for motor imagery-based brain–computer interface. Soft Comput..

[40] Jeon E., Ko W., Suk H. Domain adaptation with source selection for motor-imagery based BCI. Proceedings of the 2019 7th International Winter Conference on Brain-Computer Interface, BCI.

[41] Tang X.L., Zhang X.R. (2020). Conditional adversarial domain adaptation neural network for motor imagery EEG decoding. Entropy.

[42] Vaswani A., Shazeer N., Parmar N., Uszkoreit J., Jones L., Gomez A.N., Kaiser Ł., Polosukhin I. Attention is all you need. Proceedings of the 31st Conference on Neural Information Processing Systems (NIPS 2017).

[43] Raffel C., Shazeer N., Roberts A., Lee K., Narang S., Matena M., Zhou Y., Li W., Liu P.J. (2020). Exploring the limits of transfer learning with a unified text-to-text transformer. J. Mach. Learn. Res..

[44] Bozic V., Dordevic D., Coppola D., Thommes J., Singh S.P. (2023). Rethinking Attention: Exploring Shallow Feed-Forward Neural Networks as an Alternative to Attention Layers in Transformers. arXiv.

[45] Liu Z., Lin Y., Cao Y., Hu H., Wei Y., Zhang Z., Lin S., Guo B. Swin transformer: Hierarchical vision transformer using shifted windows. Proceedings of the IEEE/CVF International Conference on Computer Vision (ICCV).

[46] Dosovitskiy A., Beyer L., Kolesnikov A., Weissenborn D., Zhai X., Unterthiner T., Dehghani M., Minderer M., Heigold G., Gelly S. (2020). An image is worth 16x16 words: Transformers for image recognition at scale. arXiv.

[47] Zhang D., Qi T., Gao J. (2024). Transformer-based image super-resolution and its lightweight. Multimed. Tools Appl..

[48] Song Y., Zheng Q., Liu B., Gao X. (2022). EEG conformer: Convolutional transformer for EEG decoding and visualization. IEEE Trans. Neural Syst. Rehabil. Eng..

[49] Zhang D., Li H., Xie J. (2023). MI-CAT: A transformer-based domain adaptation network for motor imagery classification. Neural Netw..

[50] Li H., Zhang D., Xie J. (2023). MI-DABAN: A dual-attention-based adversarial network for motor imagery classification. Comput. Biol. Med..

[51] Song Y., Zheng Q., Wang Q., Gao X., Heng P.A. (2023). Global Adaptive Transformer for Cross-Subject Enhanced EEG Classification. IEEE Trans. Neural Syst. Rehabil. Eng..

[52] Brunner C., Leeb R., Müller-Putz G., Schlögl A., Pfurtscheller G. (2008). BCI Competition 2008—Graz Data Set A.

[53] Blankertz B., Dornhege G., Krauledat M., Müller K.R., Curio G. (2007). The non-invasive Berlin brain-computer interface: Fast acquisition of effective performance in untrained subjects. NeuroImage.

